# MicroRNA-16 is putatively involved in the NF-κB pathway regulation in ulcerative colitis through adenosine A2a receptor (A2aAR) mRNA targeting

**DOI:** 10.1038/srep30824

**Published:** 2016-08-01

**Authors:** Ting Tian, Yu Zhou, Xiao Feng, Shicai Ye, Hao Wang, Weiyun Wu, Wenkai Tan, Caiyuan Yu, Juxiang Hu, Rong Zheng, Zonghao Chen, Xinyu Pei, Hesheng Luo

**Affiliations:** 1 Department of Gastroenterology, Renmin Hospital of Wuhan University, Wuhan, 430060, China; 2 Department of Gastroenterology, The Affiliated Hospital of Guangdong Medical University, Zhanjiang, 524001, China

## Abstract

MicroRNAs (miRNAs) act as important post-transcriptional regulators of gene expression by targeting the 3′-untranslated region of their target genes. Altered expression of miR-16 is reported in human ulcerative colitis (UC), but its role in the development of the disease remains unclear. Adenosine through adenosine A2a receptor (A2aAR) could inhibit nuclear factor-kappaB (NF-κB) signaling pathway in inflammation. Here we identified overexpression of miR-16 and down-regulation of A2aAR in the colonic mucosa of active UC patients. We demonstrated that miR-16 negatively regulated the expression of the A2aAR at the post-transcriptional level. Furthermore, transfection of miR-16 mimics promoted nuclear translocation of NF-κB p65 protein and expression of pro-inflammatory cytokines, IFN-γ and IL-8 in colonic epithelial cells. Treatment with miR-16 inhibitor could reverse these effects in cells. The A2aAR-mediated effects of miR-16 on the activation of the NF-κB signaling pathway were confirmed by the A2aAR knockdown assay. Our results suggest that miR-16 regulated the immune and inflammatory responses, at least in part, by suppressing the expression of the A2aAR to control the activation of the NF-κB signaling pathway.

Inflammatory bowel disease (IBD) is a chronic and relapsing inflammatory disorder of the gastrointestinal tract that affects millions of people worldwide. IBD includes ulcerative colitis (UC) and Crohn’s disease (CD). The pathophysiology of UC remains unknown and is thought to involve disease risk genes, dysregulated gene expression and abnormal immune response to environmental triggers^1^,^2^.

Mature miRNAs are a class of small, non-coding RNA molecules that are 20–25 nucleotides in length. They bind to the 3′-untranslated region (3′-UTR) of their target messenger RNAs (mRNAs) and mediate translational repression and mRNA degradation^3^. miRNAs regulate several biological processes including cell proliferation, apoptosis, metabolism, differentiation^4^, inflammation, and immune responses by suppressing the expression of their target genes. Recent research has implicated the involvement of miRNAs in human diseases including IBD. There is increasing evidence indicating altered miRNAs expression in UC associated with the development of the disease^5^,^6^,^7^,^8^,^9^,^10^,^11^,^12^. The expression of miR-126, miR-150, and miR-155 was significantly up regulated in UC patients and in murine colitis models. These miRNAs contribute to the inflammatory reaction via regulating the NF-κB signaling pathway^8^,^9^,^10^. Activation of NF-κB signaling pathway is markedly induced in tissues affected with UC and it promotes the transcription of various pro-inflammatory cytokine genes such as interferon-gamma (IFN-γ), interleukin-1beta (IL-1β), and interleukin-8 (IL-8)^13^,^14^,^15^. Alternatively, down-regulation of certain miRNAs were reported in UC patients and in murine colitis models, such as miR-19a (targeting tumour necrosis factor-alpha (TNF-α)) and miR-192 (targeting macrophage inflammatory protein 2-alpha (MIP-2α)) in intestinal epithelial cells^11^,^12^. Further, a study using genome-wide microarray screening has identified enhanced levels of miR-16 in UC patients^5^,^12^. Earlier studies have shown that miR-16 targeted TNF-α, IL-6, cyclooxygenase-2 (COX-2), IL-12p40 and programmed cell death protein 4 (PDCD4) in cultured cell lines other than colonic epithelial cells^16^,^17^,^18^,^19^. However, the role of miR-16 in the pathophysiology of human UC is not clear.

Adenosine, a purine nucleoside, is released into the extracellular space by metabolically active cells and plays an important role in various pathophysiological processes. Adenosine regulates many biological responses including inflammation by interacting with its receptors, namely, A1, A2a, A2b, and A3^20^,^21^. Adenosine A2a receptor (A2aAR) belongs to the Gs-protein coupled receptor family and its stimulation results in the activation of adenylate cyclase^20^. A2aAR is the predominant adenosine receptor expressed in human intestinal epithelial cells^22^,^23^. High levels of extracellular adenosine lead to the activation of the A2aAR to suppress the chronic inflammation in animal colitis models^20^. *In vitro* and *in vivo* administration of a synthetic and highly selective agonist to the A2aAR inhibited inflammation via regulation of the NF-κB signaling pathway^22^,^23^,^24^. However, to date, no studies have elucidated the expression of the A2aAR and its role in the pathophysiology of human UC.

In human colonic epithelial cells, the expression of the A2bAR was found to be negatively regulated by miR-27b and miR-128a^25^. Lipopolysaccharide (LPS)-induced expression of miR-15, miR-16, and miR-214 was inversely correlated with A2aAR mRNA levels in human polymorphonuclear leukocytes (PMNs). Additionally, in PMNs, targeting sequences for miR-15 and miR-16 were identified in the 3′-UTR of the A2aAR present in a luciferase reporter vector^26^. Further studies are needed for interpreting the role of miR-16 in regulating the expression level of the A2aAR in human colonic epithelial cells and its influence on the NF-κB signaling pathway in UC.

In this study, we characterized the expression levels of miR-16 and the A2aAR in colon tissues with active UC, elucidated the regulatory role of miR-16 in the expression of A2aAR in human colonic epithelial cells, and identified the role of miR-16 and A2aAR on the NF-κB signaling pathway. We hypothesized that miR-16 may played an important role in regulating the NF-κB signaling pathway by targeting the expression of the A2aAR in human colonic epithelial cells.

## Results

### Localization of the A2aAR in human colonic mucosa

**Figure 1:**
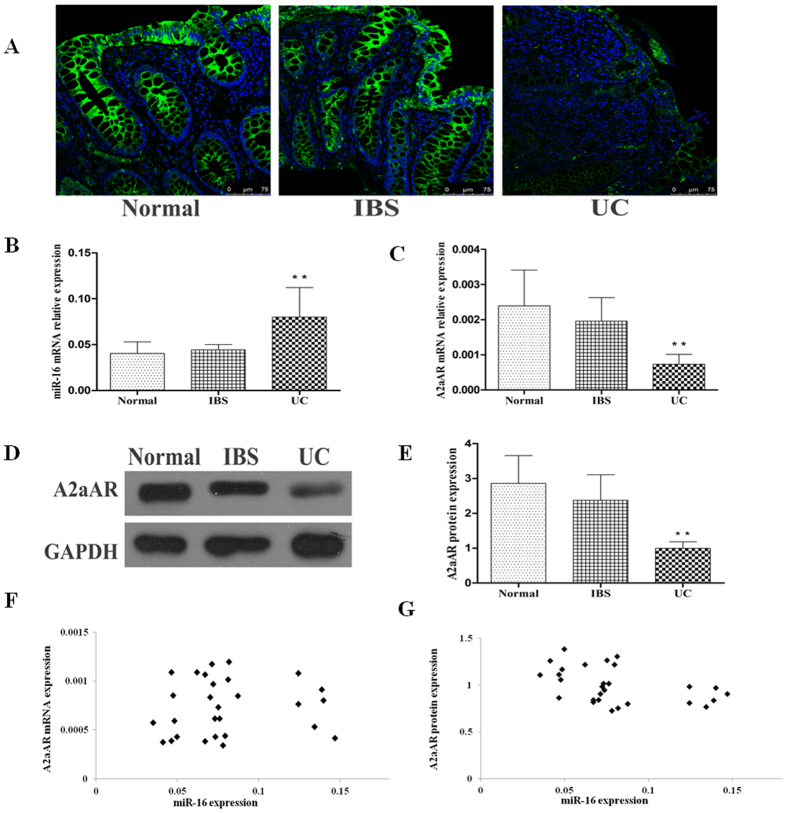
Expression of the A2aAR and miR-16 in tissues. (**A**) IF staining of sigmoid biopsy section using specific antibody to label A2aAR protein and DAPI for counterstaining the nuclei (Blue). The strong green fluorescence signal indicating A2aAR was observed mainly in the sigmoid colonic epithelial cells. The expression of A2aAR was reduced in UC inflamed tissues. (**B**) miR-16 expression, and (**C**) A2aAR mRNA expression in individual sigmoid colon tissues (normal controls n = 20, IBS n = 22 and active UC n = 28), were measured by qRT-PCR (Data are shown as mean ± SD, **P < 0.01). (**D**) A representative image of western blot for detecting A2aAR protein expression in sigmoid colon tissues. (**E**) A2aAR protein expression in sigmoid colon tissues was based on the ratio of band density of A2aAR to GAPDH protein. The band density of a western blot was measured using Quantity One software. (normal controls n = 20, IBS n = 22 and active UC n = 28. Data represent mean ± SD, **P < 0.01). (**F**) Correlation between miR-16 and A2aAR mRNA expression in active UC inflamed sigmoid tissues (n = 28, Pearson correlation *r* = 0.095, *P* > 0.05). (**G**) Correlation between miR-16 and A2aAR protein expression, miR-16 expression showed a strong negative correlation with A2aAR protein level in active UC inflamed sigmoid tissues (n = 28, Pearson correlation *r* = −0.438, *P* < 0.05).

To characterize the localization and distribution of the A2aAR in colon tissues, immunofluorescence (IF) staining was performed on sections of sigmoid biopsies from healthy subjects, irritable bowel syndrome (IBS) and active UC patients. IF results showed clear expression of the A2aAR in colonic epithelial cells (Fig. 1A). The expression of the A2aAR in colonic mucosa of IBS subjects was comparable to normal control tissues. In sigmoid colons with active UC, epithelial cells showed decreased expression of the A2aAR. These results were confirmed by the following experiments.

### Expression of miR-16 and A2aAR in sigmoid colon tissues

The expression of miR-16 and A2aAR was analysed in human sigmoid biopsies from healthy subjects, IBS, and active UC patients. Real time quantitative reverse transcription polymerase chain reaction (qRT-PCR) was used to evaluate the mRNA expression of miR-16 and A2aAR. Western blot was performed to estimate the protein content of the A2aAR. Our results indicated enhanced expression of miR-16 in sigmoid tissues of UC patients (n = 28) than IBS patients (n = 22) and normal controls (n = 20) (*P* < 0.01; Fig. 1B). Conversely, in samples with active UC, the expression of A2aAR mRNA and protein was significantly lower than the other two groups (*P* < 0.01; Fig. 1C–E). However, there was no significant difference in the expression of miR-16 and A2aAR in samples from IBS patients compared to normal controls (*P* > 0.05). The expression pattern of the A2aAR was consistent with the IF data (Fig. 1A). Furthermore, a statistically significant inverse correlation was found between the expression of miR-16 and A2aAR protein (Pearson correlation *r* = −0.438, *P* < 0.05; Fig. 1G). No significant correlation was observed between the expression of miR-16 and A2aAR mRNA levels in tissues with active UC (Pearson correlation *r* = 0.095, *P* > 0.05; Fig. 1F).

### The 3′-UTR of A2aAR is targeted by miR-16

**Figure 2:**
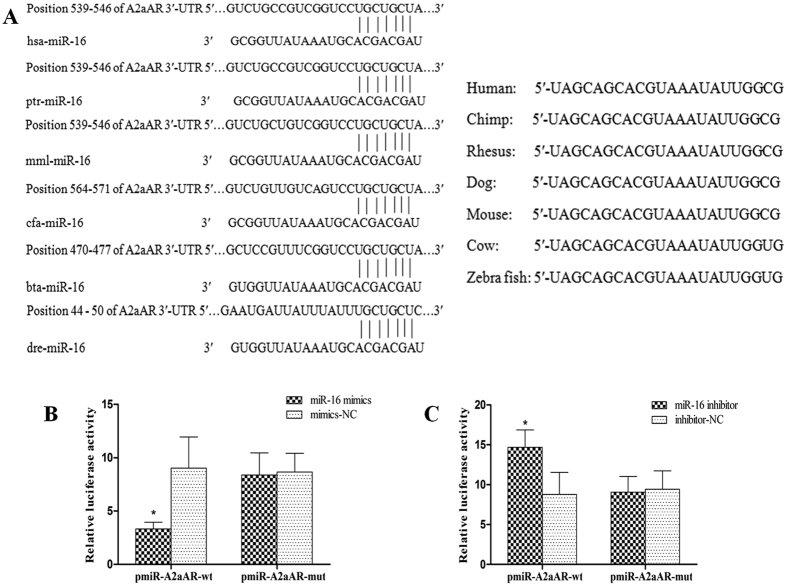
miR-16 targeting 3′-UTR of the A2aAR. (**A**) Sequence of mature miR-16 is evolutionary conserved across several species (right panel). Left panel: Positions of miR-16 target site in the 3′-UTR of A2aAR mRNA was predicted by TargetScan, including human (hsa), chimp (ptr), rhesus (mml), dog (cfa), cow (bta) and zebrafish (dre). (**B**) HT-29 cells were co-transfected with miR-16 mimics (or mimics-NC) and pmiR-A2aAR-wt (or pmiR-A2aAR-mut) vector. The relative firefly to *Renilla* luciferase activity was calculated. (**C**) HT-29 cells were co-transfected with miR-16 inhibitor (or inhibitor-NC) and pmiR-A2aAR-wt (or pmiR-A2aAR-mut) vector. The relative firefly to *Renilla* luciferase activity was calculated. Data are presented as mean ± SD of three independent experiments, *P < 0.05 as compared to other groups.

In order to predict the downstream putative targets of miR-16, *in silico* analyses was performed. The sequence of mature miR-16 was found to be evolutionary conserved across several species (Fig. 2A right panel). Additionally, miR-16 putatively targeted the 3′-UTR of A2aAR mRNA across species (except mouse), the comparative sequence alignment data of human and other species are shown in Fig. 2A (left panel). To confirm the prediction that miR-16 specifically targeted A2aAR mRNA, we constructed luciferase reporter vectors carrying the wild type sequence of the A2aAR 3′-UTR downstream of the luciferase gene (named as pmiR-A2aAR-wt), and the corresponding mutant (named as pmiR-A2aAR-mut, detailed in M&M section). HT-29 cells were co-transfected with miR-16 mimics and pmiR-A2aAR-wt or pmiR-A2aAR-mut and a luciferase reporter assay was performed. Our results indicated significant reduction in the luciferase activity of pmiR-A2aAR-wt by miR-16 mimics in HT-29 cells compared to cells transfected with miRNA mimics negative control (mimics-NC) (*P* < 0.05; Fig. 2B). Additionally, transfection with miR-16 inhibitor (blocking the activity of miR-16) significantly increased the luciferase activity of pmiR-A2aAR-wt in co-transfected HT-29 cells compared to cells transfected with miRNA inhibitor negative control (inhibitor-NC) (*P* < 0.05; Fig. 2C). Luciferase activity of pmiR-A2aAR-mut in co-transfected HT-29 cells was not altered by transfection with either miR-16 mimics or miR-16 inhibitor (*P* > 0.05; Fig. 2B,C). Collectively, these data confirm that the A2aAR is a direct target of miR-16 in colonic epithelial cells.

### Endogenous expression of A2aAR is regulated by miR-16

**Figure 3:**
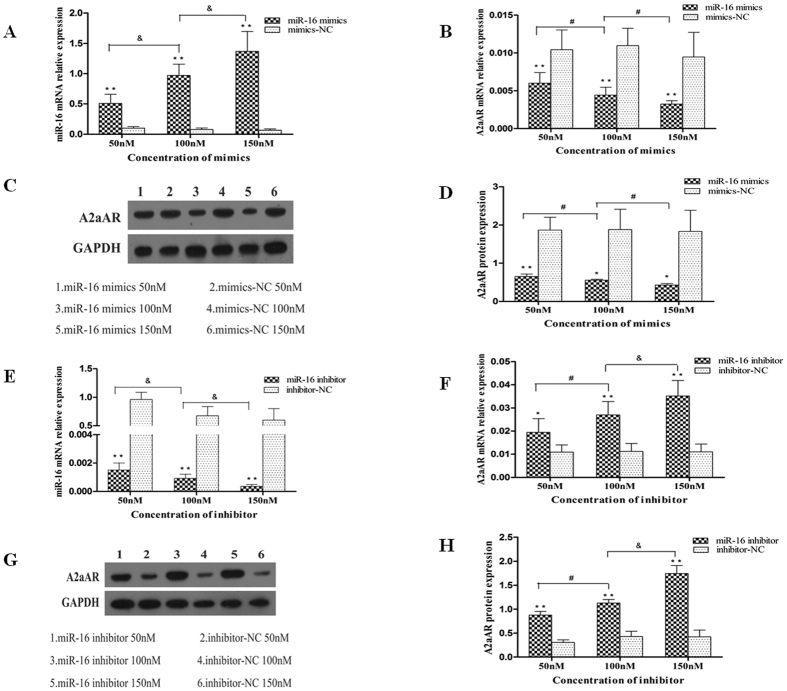
miR-16 regulates the endogenous expression of A2aAR. (**A**) qRT-PCR detected enhanced expression of miR-16 in a dose-dependent manner (&*P* < 0.01) in HT-29 cells transfected with miR-16 mimics compared to cells transfected with mimics-NC (**P < 0.01). (**B**) Expression of the A2aAR mRNA measured by qRT-PCR and (**C**) the expression of A2aAR protein detected by western blot. (**D**) Quantification of western blot by Quantity One software indicating decreased A2aAR protein expression in HT-29 cells transfected with miR-16 mimics compared to the relevant mimics-NC groups (**P < 0.01, *P < 0.05), in a dose-dependent manner (#*P* < 0.05). (**E**) miR-16 expression was reduced in HT-29 cells transfected with miR-16 inhibitor compared to cells transfected with inhibitor-NC groups (**P < 0.01), in a dose-dependent manner (&*P* < 0.01). The expression of (**F**) A2aAR mRNA and (G,H) protein was increased in HT-29 cells transfected with miR-16 inhibitor compared to cells transfected with the inhibitor-NC (**P < 0.01, *P < 0.05), in a dose-dependent manner (^&^*P* < 0.01, ^#^*P* < 0.05).

To investigate the role of miR-16 in the regulation of the endogenous expression of the A2aAR, miR-16 mimics and mimics-NC were transiently transfected into HT-29 cells at 50 nM, 100 nM, and 150 nM, respectively. Results from qRT-PCR indicated enhanced expression of mature miR-16 (*P* < 0.01; Fig. 3A) and decreased expression of A2aAR mRNA (*P* < 0.05; Fig. 3B) in a dose dependent manner in cells transfected with miR-16 mimics compared to the mimics-NC group. Protein levels of the A2aAR mimicked the expression pattern of its mRNA in the above cells (*P* < 0.05; Fig. 3B–D). Conversely, in cells transfected with miR-16 inhibitor, the expression of mature miR-16 was significantly decreased (*P* < 0.01; Fig. 3E), while the expression of A2aAR mRNA and protein increased more than 50% compared to cells transfected with inhibitor-NC (*P* < 0.05; Fig. 3F–H). These results indicate that miR-16 could negatively regulate the endogenous expression of the A2aAR in colonic epithelial cells.

### Effects of miR-16 on the activation of the NF-κB signaling pathway

**Figure 4:**
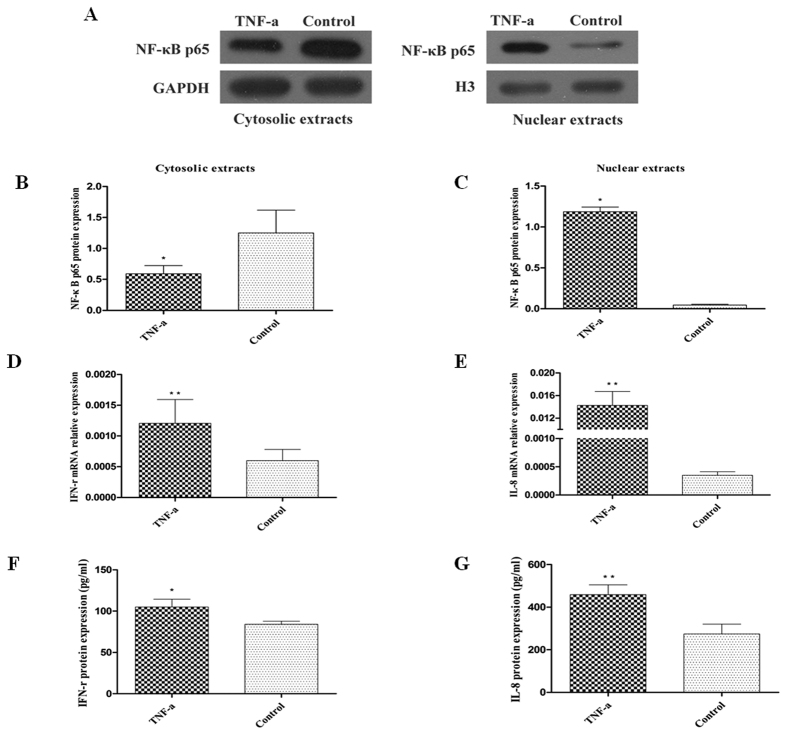
Effect of TNF-α stimulation on NF-κB p65 activation and IFN-γ and IL-8 expression. HT-29 cells were treated with or without TNF-α for 12 h or 24 h. (**A–C**) NF-κB p65 protein in cytosolic and nuclear fraction of these cells were analysed by western blot, and quantified by Quantity One software. (**D,E**) IFN-γ and IL-8 mRNA expression in HT-29 cells detected by qRT-PCR. (**F,G**) ELISA for IFN-γ and IL-8 in supernatants released by these cells. Data are presented as mean ± SD, *P < 0.05, **P < 0.01 compared to untreated controls.

Upon cell stimulation and activation of NF-κB signaling pathway, NF-κB p65 is translocated from the cytoplasm to the nucleus. In order to study the relation between miR-16 and NF-κB signaling pathway, activation of the NF-κB signaling pathway was first validated in HT-29 cells that were stimulated with TNF-α. Western blot analysis of cytosolic and nuclear protein fractions indicated localization of NF-κB p65 protein in the cytoplasm of untreated cells and in the nuclear protein fraction in TNF-α stimulated cells (*P* < 0.05; Fig. 4A–C). This confirmed the translocation of NF-κB p65 protein from the cytoplasm to the nucleus upon stimulation by TNF-α in HT-29 cells. Additionally, levels of the pro-inflammatory cytokines IFN-γ and IL-8 were significantly increased in TNF-α-treated cells compared to untreated cells (*P* < 0.05; Fig. 4D–G).

**Figure 5:**
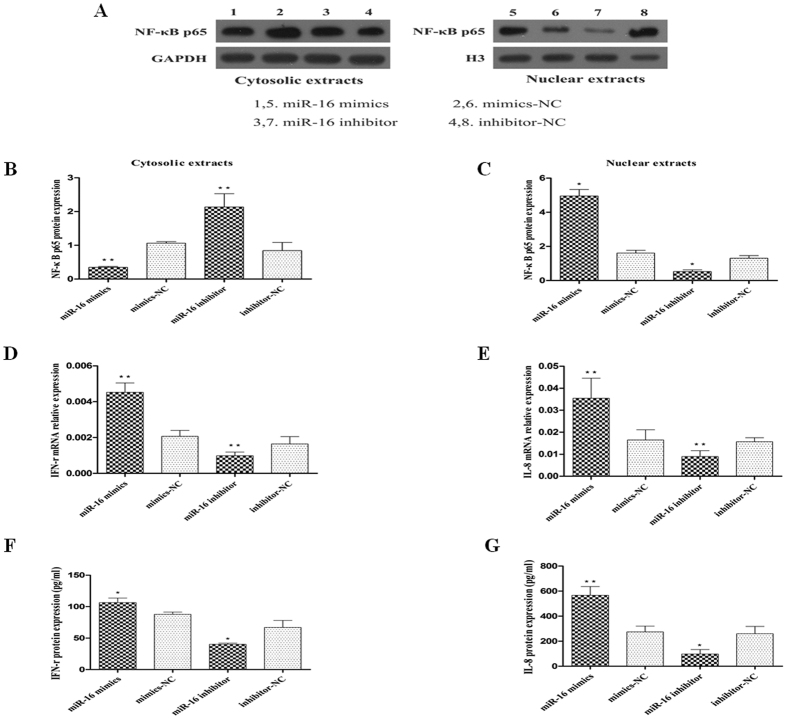
Effect of miR-16 on the activation of NF-κB p65 and expression of IFN-γ and IL-8. HT-29 cells were transfected with 150 nM of miR-16 mimics, mimics-NC, miR-16 inhibitor and/or inhibitor-NC, respectively. These cells were then treated with TNF-α. (**A–C**) NF-κB p65 protein in cytosolic and nuclear fraction of these cells as analysed by western blot and quantified. Compared to the corresponding NC control, nuclear translocation of NF-κB p65 protein was enhanced in cells transfected with miR-16 mimics, and decreased in cells transfected with miR-16 inhibitor. (**D,E**) qRT-PCR detecting the expression of IFN-γ and IL-8 mRNAs in these cells. (**F,G**) ELISA for IFN-γ and IL-8 in cell supernatants. Data are presented as mean ± SD, *P < 0.05, **P < 0.01 compared to the corresponding NC control.

To study the effects of miR-16 on the activation of the NF-κB signaling pathway, 150 nM of miR-16 mimics or mimics-NC were transiently transfected into HT-29 cells. The transfected cells were then stimulated with TNF-α and analysed. The expression of NF-κB p65 protein was significantly decreased in the cytoplasm and increased in the nucleus in TNF-α treated miR-16 mimics transfected cells compared to cells transfected with mimics-NC (*P* < 0.05; Fig. 5A–C). Accordingly, the expression of IFN-γ and IL-8 mRNAs and proteins were increased in miR-16 mimics transfected cells compared to cells transfected with mimics-NC (*P* < 0.05; Fig. 5D–G).

The activation of the NF-κB signaling pathway was investigated by transfecting TNF-α-treated HT-29 cells with 150 nM of miR-16 inhibitor or inhibitor-NC. Compared to inhibitor-NC, miR-16 inhibitor increased the localization of NF-κB p65 protein in the cytoplasm and decreased it in the nucleus (*P* < 0.05; the right two bars on Fig. 5A–C). Correspondingly, the expression of IFN-γ and IL-8 was significantly decreased in cells transfected with miR-16 inhibitor compared to cells transfected with inhibitor-NC (*P* < 0.05; the right two bars on Fig. 5D–G). These results representing gain-of-function and loss-of-function indicate that miR-16 influenced the activation of the NF-κB signaling pathway and pro-inflammatory cytokine production in colonic epithelial cells.

### The A2aAR mediates the effects of miR-16 on the activation of the NF-κB signaling pathway

**Figure 6:**
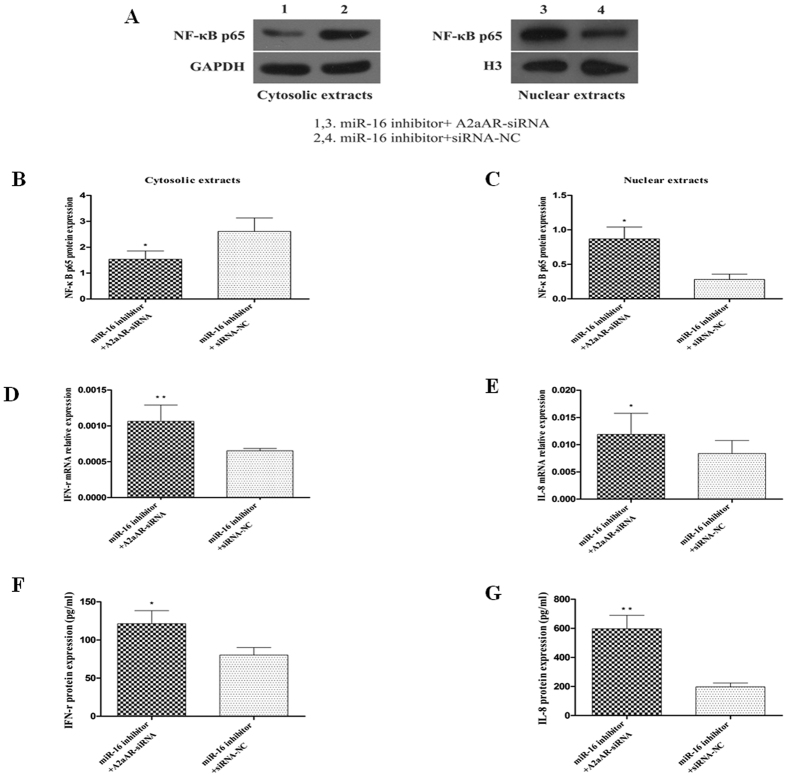
Effect of A2aAR-siRNA on NF-κB p65 activation and IFN-γ and IL-8 expression. HT-29 cells were co-transfected with 150 nM of miR-16 inhibitor and A2aAR-siRNA or siRNA-NC, and stimulated with TNF-α. (**A–C**) Western blot analyses and quantification of NF-κB p65 protein in cytosolic and nuclear fraction of cells. (**B**) Silencing of the A2aAR significantly decreased the NF-κB p65 protein expression in cytosolic extracts, (**C**) and increased the NF-κB p65 protein expression in nuclear extracts. (**D,E**) Expression of IFN-γ and IL-8 mRNAs in these cells as detected by qRT-PCR. (**F,G**) ELISA for IFN-γ and IL-8 present in cell supernatants. Data are presented as mean ± SD, *P < 0.05, **P < 0.01 compared to cells transfected with siRNA-NC.

Our results indicated that miR-16 suppressed the expression of the A2aAR and increased the activation of the NF-κB signaling pathway. Accordingly, inhibition of miR-16 by its inhibitor increased the expression of the A2aAR and decreased the activation of the NF-κB signaling pathway. To investigate whether the effects of miR-16 on the activation of the NF-κB signaling pathway were though regulating the expression of the A2aAR. HT-29 cells were analysed upon treatment with miR-16 inhibitor (increases the expression of the A2aAR) followed by treatment with siRNA against the A2aAR (knocks-down the expression of the A2aAR). Results were evaluated to determine if knocking down the expression of the A2aAR could reverse the effects of miR-16 inhibitor on the NF-κB signaling pathway. Results of qRT-PCR confirmed down regulation of the expression of A2aAR mRNA in cells transfected with A2aAR-siRNA compared to cells transfected with siRNA negative control (siRNA-NC) (data not shown). Co-transfection of miR-16 inhibitor (150 nM) with A2aAR-siRNA (150 nM) in TNF-α-treated HT-29 cells, resulted in increased translocation of NF-κB p65 protein into the nuclear area, and higher production of IFN-γ and IL-8 compared to cells co-transfection with miR-16 inhibitor and siRNA-NC (*P* < 0.05; Fig. 6A–G). These results indicate that knocking down the expression of the A2aAR by siRNA could reverse the effects of miR-16 inhibitor on the activation of the NF-κB signaling pathway stimulated by TNF-α. Taken together, our results suggest that the effects of miR-16 on the activation of the NF-κB signaling pathway might be mediated by the A2aAR.

## Discussion

Recent studies have demonstrated the involvement of miRNAs in the pathophysiology of inflammatory diseases, including IBD. They provided new insights in understanding the pathogenesis of these diseases. A genome-wide microarray screening study had identified enhanced expression of miR-16 in UC patients^5^,^12^. In the present study, we specifically analysed the expression of miR-16 in individual sigmoid biopsies from clinical subjects. Consistent with previous studies, our results indicated enhanced expression of miR-16 in active UC patients compared to IBS patients and healthy volunteers. These results suggest that altered expression of miR-16 might be associated with IBD. However, the role of miR-16 in the development of UC has not been elucidated.

miR-16 is known to suppress TNF-α, IL-6 and COX-2 expression in tumour cell lines like HeLa cells^16^, and in human THP-1 monocytic cells treated with S100b^17^. Recently, a study demonstrated that miR-16 mimics could directly target PDCD4 in macrophage-derived foam cells and could suppress the production of pro-inflammatory cytokines^19^. The 3′-UTR of the A2aAR in a luciferase reporter vector was targeted by miR-15 together with miR-16 in human PMNs, but not in T cells^26^. These results indicate the role of miR-16 in inflammation and immune response that are mediated by targeting different genes in a tissue and cell specific manner.

The A2aAR regulates many biological responses, including inflammation. Activation of the A2aAR suppressed chronic inflammation in animal colitis models^20^,^27^,^28^,^29^. To date, there exists only a single report related to the expression of the A2aAR in IBD associated human colonic mucosa, focusing on 22 purine-related genes re-analysed using the gene expression profiles deposited in NCBI GEO databank. This study reported enhanced the expression of the A2aAR mRNA in sigmoid biopsies with active CD (n = 11), while no change was detected in UC patients (n = 10)^30^. Validation by an alternative approach was lacking in this report. Hence, we investigated the role of the expression of the A2aAR in human colonic mucosa in UC. Results of our study indicated reduced expression of the A2aAR in sigmoid colonic mucosa in active UC patients (n = 28) compared to normal controls (n = 20). Protein expression of the A2aAR showed inverse correlation with the expression of miR-16 in sigmoid mucosa in active UC patients (Fig. 1B,E,G). According to the data from *in silico* analysis, mature miR-16 sequences were evolutionary conserved across several species and they targeted the corresponding 3′-UTR of A2aAR mRNA (Fig. 2A). Luciferase reporter assay indicated that miR-16 could directly and specifically target 3′-UTR of the A2aAR. Further, using gain-of-function (using miR-16 mimics) and loss-of-function (using miR-16 inhibitor) studies, we demonstrated that miR-16 inhibited the endogenous expression of the A2aAR at the post-transcriptional level in colonic epithelial cells (HT-29 cells). It worth to note, using approaches such as Argonaute co-precipitating-complex assay to precisely determine the direct interaction between miR-16 and A2aAR mRNA *in vivo* would be greatly appreciated in the future.

NF-κB molecules belong to a family of inducible transcription factors, including p65 (RelA), NF-κB p65 is activated by external and/or internal stimuli and is translocated from the cytoplasm to the nucleus, where it triggers the transcription of response genes. NF-κB plays a pivotal role in the initiation and perpetuation of an inflammatory/immune response by promoting the expression of major inflammatory mediators such as cytokines, chemokines, and adhesion molecules^14^,^31^. Several studies suggest NF-κB as a key modulator in governing the molecular network leading to various cellular functions associated with IBD^8^,^32^,^33^. Previous studies have demonstrated that the activation of the A2aAR could inhibit the NF-κB signaling pathway in LPS-induced smooth muscle cells^34^ and immune cells^35^. An *in vivo* mouse model demonstrated that the activation of the A2aAR could inhibit osteoclast differentiation and regulate the bone turnover via PKA-dependent inhibition of nuclear translocation of NF-κB. These studies demonstrate that A2aAR can regulate the NF-κB signaling pathway not only *in vitro*, but also *in vivo*, suggesting that they could be the target in inflammatory diseases such as rheumatoid arthritis^24^.

To determine whether miR-16 could affect the NF-κB signaling pathway in colonic epithelial cells, miR-16 mimics or miR-16 inhibitor was transfected into TNF-α-treated HT-29 cells. Enhanced cellular expression of miR-16 using miR-16 mimics promoted the nuclear translocation of NF-κB p65 leading to the activation of the NF-κB signaling pathway associated with enhanced expression of IFN-γ and IL-8. Conversely, transfection with miR-16 inhibitor resulted in enhanced expression of the A2aAR and decreased activation of the NF-κB signaling pathway (Fig. 5). Our results are consistent with a recent report^19^. Further, we aimed to determine whether the effects of miR-16 on NF-κB signaling pathway were regulated by the expression of A2aAR. We co-transfected HT-29 cells with miR-16 inhibitor and A2aAR-siRNA (knockdown A2aAR expression). This co-transfection resulted in enhanced translocation NF-κB p65 into the nucleus and increased production of IFN-γ and IL-8 when stimulated with TNF-α, indicating that A2aAR-siRNA reversed the effects of miR-16 inhibitor on the NF-κB signaling pathway. Recently, an interesting study was performed in a murine colitis model to evaluate the therapeutic effects of using miR-16 precursors conjugated with colonic macrophage targeting vectors (based on galactosylated low molecular weight chitosan). The results indicated significant up-regulation of miR-16 in colonic macrophages associated with decreased expression of TNF-α and IL-12p40, specifying its role in suppressing the associated mucosal inflammation^18^.

Taken together, we demonstrated that miR-16 inhibited the expression of A2aAR protein at the post-transcriptional level and promoted activation of the NF-κB signaling pathway. Our results imply regulation of activation of the NF-κB signaling pathway by miR-16, at least in part, by targeting the expression of the A2aAR. Based on our findings and those from others, both miR-16 and A2aAR are considered as potential therapeutic targets in controlling inflammation and would benefit IBD patients.

## Materials and Methods

### Human tissue samples

Colonoscopic pinch biopsies from the sigmoid colon of 70 patients undergoing screening colonoscopies were obtained from healthy subjects (n = 20) and patients with IBS (n = 22) or with active UC (n = 28) at the Affiliated Hospital of Guangdong Medical University, Zhanjiang, China. Biopsies from all patients were assessed between July 2011 and September 2012. Rome III criteria were used to diagnose IBS^36^. Recurrent abdominal pain or discomfort at least 3 days per month in the last 3 months, associated with two or more of the following: 1. Improvement with defecation; 2. Onset associated with a change in the frequency of stool; 3. Onset associated with a change in the form of stool. To separate these chronic conditions from transient gut symptoms, they must have occurred for the last 3 months, with the onset of symptoms at least 6 months prior to diagnosis, and no evidence of an inflammatory, anatomic, metabolic, or neoplastic process that explains the subject’s symptoms. Colonoscopic pinch biopsies from sigmoid colons were obtained from those subjects who have not received any treatment for at least 2 months before colonoscopy. The diagnosis of UC was based on standard clinical, histological criteria, and colonoscopy. The criteria for inclusion were new onset of active UC before initiation of anti-inflammatory treatment. Biopsies were taken from the inflamed sites of sigmoid colon. Disease activities were determined using the ulcerative colitis disease activity index (UCDAI)^37^, which is a series of qualifiers related to symptoms of UC including stool frequency, rectal bleeding, appearance of the lining of the colon, and a physician rating of the disease activity. Each of these items was given a number from 0 to 3, with 3 being the highest for rating a disease activity. Ratings of 0–2 were recorded as remission, 3–6 as mild, 7–10 as moderate, >10 as severe UC, respectively. Patients with UCDAI >7 were included in the study. The biopsies were immersed in paraformaldehyde prior to paraffin embedding, or snap frozen in liquid nitrogen and stored at −80 °C until use. The clinical characteristics of each patient group are listed in Table 1.

### Immunofluorescence (IF)

IF staining was performed according to the standard protocol using 5-μm-thick transverse-cut paraffin sections from sigmoid colon biopsies. Briefly, paraffin sections were rendered permeable by incubation in 0.1% Triton X-100 for 30 min, blocked with 10% donkey serum albumin at room temperature (RT) for 30 min, followed by incubation with rabbit anti-human A2aAR primary antibody (1:30 dilution, Santa Cruz Biotechnology) overnight at 4 °C. After rinsing three times with phosphate-buffered saline (PBS), the sections were incubated with secondary antibody (Donkey anti-rabbit FITC, 1:100 dilution, Santa Cruz Biotechnology) for 1 h at RT in the dark, and were washed three times with PBS. Negative controls were prepared by omitting the primary antibody. Nuclei were counterstained with DAPI and the coverslips were mounted on slides by using a fluorescent mounting medium. The stained sections were evaluated using a Leica inverted fluorescence microscope.

### Real-time quantitative reverse transcriptase polymerase chain reaction (qRT-PCR)

For qRT-PCR experiments, total RNA from colonic biopsies or cultured cells were extracted using RNAiso Plus according to the manufacturer’s instructions (Takara, Japan).The concentration and purity of the isolated total RNA samples were verified using an Eppendorf^®^ BioPhotometer Plus (Eppendorf, Hamburg, Germany). The One Step PrimeScript^®^ miRNA cDNA Synthesis Kit (Takara, Japan) and PrimeScript^®^ RT Master Mix Perfect Real Time (Takara, Japan) were used to reverse-transcribe the miRNA and mRNA, respectively, according to the manufacturer’s instructions. qRT-PCR reactions were performed in triplicate using SYBR^®^ Premix Ex Taq^TM^ II (Perfect Real Time, Takara, Japan) on LightCycler^®^ (Roche Diagnostics, Nutley, NJ, USA) in a 96-well format, over 45 cycles with denaturation at 95 °C for 10 s and annealing at 60 °C for 20 s. U6 small RNA was used to normalize the levels of miR-16, and GAPDH was used to normalize the mRNA expression levels of A2aAR, IFN-γ, and IL-8. The relative expression was calculated using the 2^−∆CT^ method. For performing miRNA qRT-PCR, the reverse primer was included in the kit. Forward miRNA primers and mRNA primers were purchased from Sangon Biotech (Shanghai, China). The sequences of these primers are listed in Table 2.

### Cell culture and reagents

HT-29 cells were cultured in RPMI-1640 medium (Gibco, USA), supplemented with 10% foetal bovine serum (FBS; Gibco, USA), 100 IU/mL penicillin, and 100 μg/mL streptomycin in a 5% CO_2_ incubator at 37 °C. miR-16 mimics (double stranded 5′-UAGCAGCACGUAAAUAUUGGCG-3′/3′-AUCGUCGUGCAUUUAUAACCGC-5′), a universal miRNA mimics negative control (mimics-NC), miR-16 inhibitor (antisense oligonucleotides 5′-CGCCAAUAUUUACGUGCUGCUA-3′), a universal miRNA inhibitor negative control (inhibitor-NC), A2aAR-siRNA (5′-CAACUACUUUGUGGUGUCA-dTdT-3′/3′- dTdT-GUUGAUGAAACACCACAGU-5′), and a universal siRNA-negative control (siRNA-NC) were purchased from RiboBio (Guangzhou, China). Both mimics-NC and inhibitor-NC were mixed *Caenorhabditis elegans* miRNAs without any overlapping with human or mouse miRNAs.

### MiRNA mimics or inhibitor transfection

HT-29 cells were cultured in 6- or 12-well plates till they reached 90% confluence and were incubator overnight at 37 °C with 5% CO_2_. Different concentrations (50 nM, 100 nM, and 150 nM) of miR-16 mimics, miR-16 inhibitor, or negative controls were transfected into HT-29 cells, using lipofectamine™ 2000 reagent (Invitrogen, USA) in Opti-MEM (Gibco, USA), according to the manufacturer’s instructions. The total cellular RNA and proteins were harvested separately, and stored at −80 °C until used.

### Identification of potential downstream targets of miR-16

To predict potential targets of miR-16, three *in silico* analysis programs were used for microRNA targets prediction: Micro Cosm Targets (http://www.ebi.ac.uk/enright-srv/microcosm/htdocs/targets/v5/), TargetScan (http://www.targetscan.org/) and PicTar (http://pictar.mdc-berlin.de/). The analysis revealed the presence of a putative miRNA-16 binding site in the 3′-UTR of A2aAR mRNA (RefSeq NM_000675).

### Transfection of A2aAR 3′-UTR construct and luciferase report assay

The human A2aAR 3′-untranslated region (3′-UTR), generated by PCR amplification, was cloned downstream of Renilla luciferase of pmiR-RB-REPORT™ dual luciferase reporter plasmid (RiboBio Co. Ltd., China). The PCR primers selected were: A2aAR-3′UTR-wt-F, 5′-GCGGCCTCGAGTCCTGATGATTCATGGAGTTT-3′; A2aAR-3′UTR-wt-R, 5′-AATGCGGCCGCTCAGACGCCATTCTCATTT-3′; A2aAR-3′UTR-mut-F, 5′-GTCGGTCCACGACGATACCTGGCACCAGAGCCTC-3′; and A2aAR-3′UTR-mut-R, 5′-TGCCAGGTATCGTCGTGGACCGACGGCAGACCCA-3′(underlined for XhoI and NotI sites). These constructs containing the wild type sequence of A2aAR 3′-UTR was named as pmiR-A2aAR-wt. A mutant (named as pmiR-A2aAR-mut) was created by site-directed mutagenesis to replace the miR-16 binding site TGCTGCTA with ACGACGAT. To determine the specific effect of miR-16 on the 3′-UTR of A2aAR, HT-29 cells were cultured in 24-well plates and co-transfected with 500 ng of pmiR-A2aAR-wt or pmiR-A2aAR-mut, 150 nM miR-16 mimics, or miR-16 inhibitor, or negative controls using the lipofectamine™ 2000 reagent. Renilla and Firefly luciferase levels were measured at 48 h post-transfection using the Dual-Luciferase Reporter Assay System (Promega, USA), according to the manufacturer’s instructions. The tests were performed in triplicate.

### TNF-*α* treatment

HT-29 cells were cultured in 6- or 12-well plates until 90% confluence and incubated overnight at 37 °C and 5% CO_2_. The cells were transfected with 150 nM of miR-16 mimics, or miR-16 inhibitor, or negative controls. Further, cells were co-transfected with 150 nM miR-16 inhibitor and A2aAR-siRNA or siRNA-NC (150 nM), using the lipofectamine™ 2000 reagent. After 48 h, the cells were stimulated with TNF-α (10 ng/mL) for 12 h and 24 h. The total RNA, protein, and media were collected.

### Western blot analysis

Total protein from sigmoid colon biopsies and HT-29 cells with various treatments were isolated using cell lysis buffer containing phenylmethylsulfonyl fluoride (Beyotime, China). The cytoplasmic and nuclear protein extract of HT-29 cells with various treatments was separately collected using a Sangon Biotech Kit (Shanghai, China), according to the manufacturer’s instructions. Protein concentrations were determined using a BCA protein assay kit (Beyotime, China). Equal amounts of protein were separated on 12% SDS-polyacrylamide gel electrophoresis (SDS-PAGE) and transferred onto a polyvinylidene fluoride membrane (PVDF; Millipore, Billerica, MA, USA). The membranes were blocked for 1 h with 5% fat-free milk at RT, incubated with primary antibodies against A2aAR (rabbit anti-human, 1:600 dilution, Santa Cruz Biotechnology), NF-κB P65 (mouse anti-human, 1:500 dilution, Santa Cruz Biotechnology), GAPDH (mouse anti-human, 1:1000 dilution, Beyotime, China), and H3 (rabbit anti-human, 1:1000 dilution, Beyotime, China), at 4 °C overnight. Membranes were washed three times with Tris-buffered saline with Tween-20 (TBST), and incubated with the corresponding secondary antibodies (HRP-labelled Goat Anti-rabbit IgG, HRP-labelled Goat Anti-mouse IgG, 1:1000; Beyotime, China) for 1 h at RT. Signals were detected with an electrochemiluminescence (ECL) detection reagent (Beyotime, China). The images were obtained on Kodak film and quantified using Quantity One software (Bio-Rad, Hercules, CA, USA).

### Enzyme-linked immunosorbent assay (ELISA)

ELISA was used to measure the IFN-γ and IL-8 protein in the media of HT-29 cells. Cell supernatants were collected after various treatments and cleared by centrifugation in order to remove the cell debris. The concentrations of IFN-γ and IL-8 were determined through ELISA kits, according to the manufacturer’s instructions (Sangon, China), using an ELISA plate reader. For each experiment, three individual wells of each molecule concentration were prepared. The concentrations of IFN-γ and IL-8 released into the medium are expressed in pg/mL.

### Statistical analysis

Each experiment was performed in triplicate, as stated. Data are expressed as mean ± standard deviation (SD). Multiple groups were compared by one-way analysis of variance (ANOVA) and two groups were determined using a *t*-test. SPSS 19.0 software (SPSS Inc. USA) was used for the analyses and differences were considered statistically significant at *P* < 0.05.

### Ethical Statement

The Institutional Review Board of the Affiliated Hospital of Guangdong Medical University approved the study. All patients gave written informed consent for their participation. The methods were carried out in accordance with the university’s scientific research guidelines and regulations.

## Additional Information

**How to cite this article**: Tian, T. *et al*. MicroRNA-16 is putatively involved in the NF-κB pathway regulation in ulcerative colitis through adenosine A2a receptor (A2aAR) mRNA targeting. *Sci. Rep.* **6**, 30824; doi: 10.1038/srep30824 (2016).

## Figures and Tables

**Table 1:** Clinical Characteristics of Patients.

	Normal	IBS	UC
No. of patients	20	22	28
Gender (male/female)	9/11	10/12	15/13
Age (yrs)			
Mean ± SD	40 ± 13.2	48.3 ± 11.7	43.5 ± 16.4
Range	28–67	30–65	21–78
DAI score			
Mean ± SD	—	—	8.5 ± 1.4
Range	—	—	7–10
Duration of symptoms (yrs)			
Mean ± SD	—	4 ± 1.2	3 ± 1.0
Range	—	18	0.5–6

**Table 2:** Primers used for quantitative real-time PCR in this study.

Name	Direction	Primer (5′-3′)
For microRNA qPCR		
Universal qPCR primer	Reverse	The One Step PrimeScript^®^ miRNA cDNA Synthesis Kit (Takara)
miR-16	Forward	TAGCAGCACGTAAATATTGGCG
U6 snRNA	Forward	CTCGCTTCGGCAGCACA
For mRNA qPCR		
A2aAR	Forward	AAGGAGGGCAAGAACCACTC
	Reverse	AGCACACAGGCAAAGAAGTTG
IFN-γ	Forward	AATGTCCAACGCAAAGCAAT
	Reverse	AGCATCTGACTCCTTTTTCGC
IL-8	Forward	GCAGAGGGTTGTGGAGAAGT
	Reverse	AACCCTACAACAGACCCACA
GAPDH	Forward	GGGTGTGAACCATGAGAAGT
	Reverse	CAGTGATGGCATGGACTGTG
